# Supplementary material for: Magnetic field-tuned Fermi liquid in a Kondo insulator
Source: Nat Commun. 2019 Dec 2;10:5487. doi: 10.1038/s41467-019-13421-w (PMC6889157; doi:10.1038/s41467-019-13421-w)
Supplement: Supplementary file 1 — Supplementary Information [file 41467_2019_13421_MOESM1_ESM.pdf]

Magnetic field-tuned Fermi liquid in a Kondo insulator, Kushwaha *et al.*, Supplementary Figures.

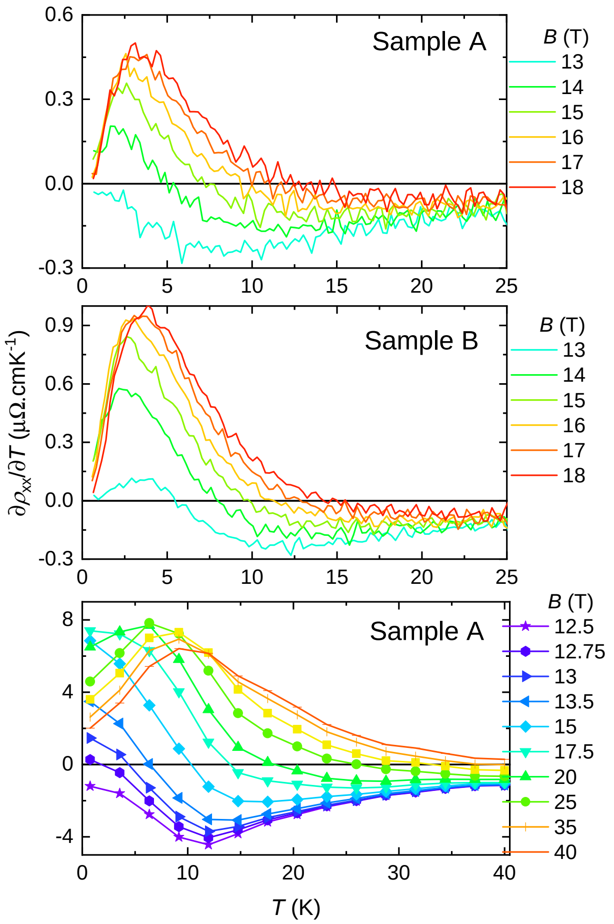

Supplementary Figure 1: Derivatives of the electrical resistivity of $Ce_3Bi_4Pd_3$. The derivatives $\partial\rho_{xx}/\partial T$ of the electrical resistivity of $Ce_3Bi_4Pd_3$ of sample A in static fields, sample B in static fields and sample A in pulsed fields. The actual resistivity data are shown in Fig. 4 of the main manuscipt.

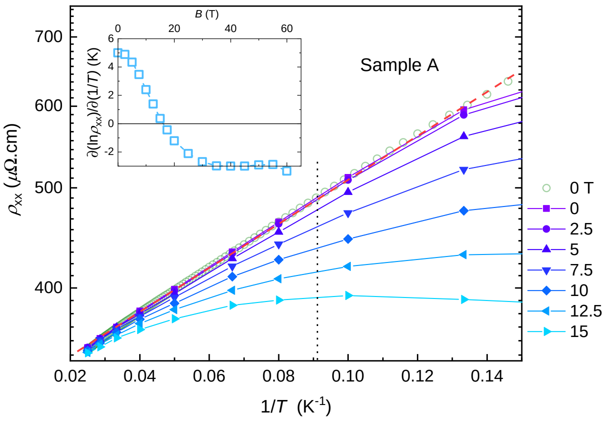

Supplementary Figure 2: Field-dependent Arrhenius plots of $Ce_3Bi_4Pd_3$. Logarithmically-scaled electrical resistivity of $Ce_3Bi_4Pd_3$ sample A plotted versus $1/T$ and different magnetic fields as indicated. The inset shows the slope as a function of magnetic field at 11 K, thus providing electrical transport evidence for the closure of the Kondo gap.

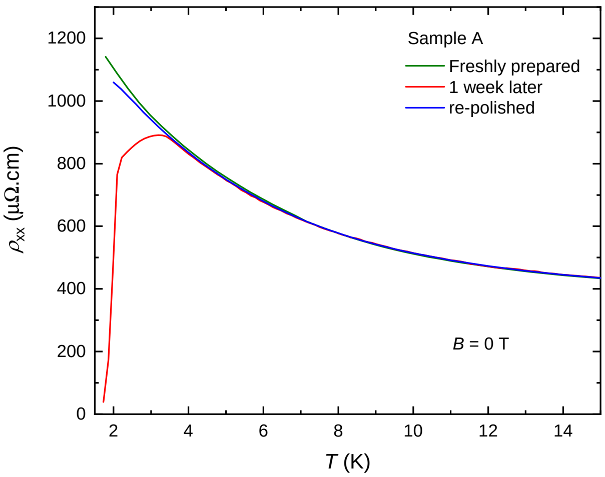

Supplementary Figure 3: Electrical resistivity of $Ce_3Bi_4Pd_3$. The electrical resistivity of $Ce_3Bi_4Pd_3$ (sample A), after being freshly prepared (green curve), thermally cycled (red curve) and re-polished to remove surface contamination (blue curve).

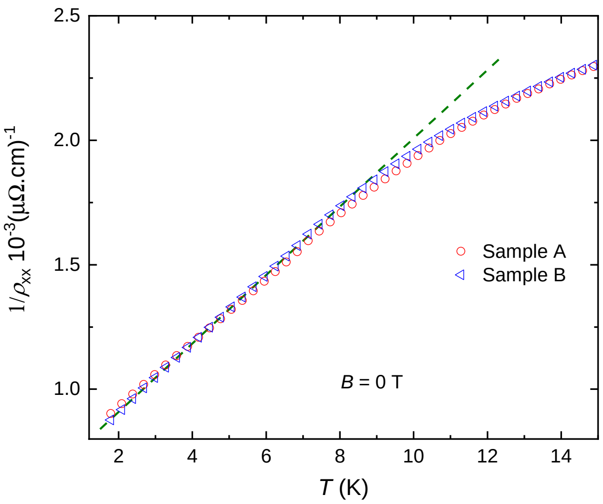

Supplementary Figure 4: Low temperature reciprocal resistivity of $Ce_3Bi_4Pd_3$. Low temperature reciprocal resistivity of $Ce_3Bi_4Pd_3$ samples A and B (in difference colors as indicated), indicating a linear-in-T component.

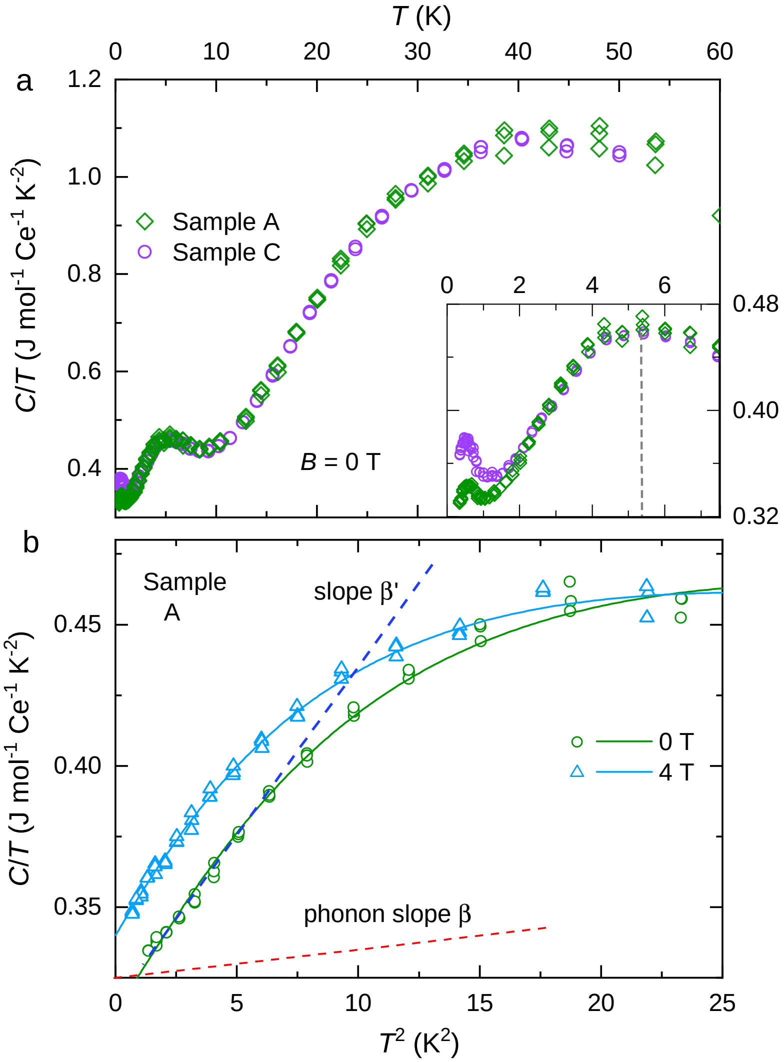

Supplementary Figure 5: Heat capacity of $Ce_3Bi_4Pd_3$. a, Heat capacity divided by temperature C/T plotted versus T for two samples A and C (in different colors as indicated). The inset shows an expanded view of the low temperature region. **b**, Low temperature portion of C/T (from Fig. 6 of the main text) plotted versus T^2 (symbols and colors refer to different fields as indicated, while lines refer to fits to the Schotte-Schotte model), showing an approximate T^2 contribution at low T on top of that βT^2 due to phonons (the assumed phonon contribution is shown in red).

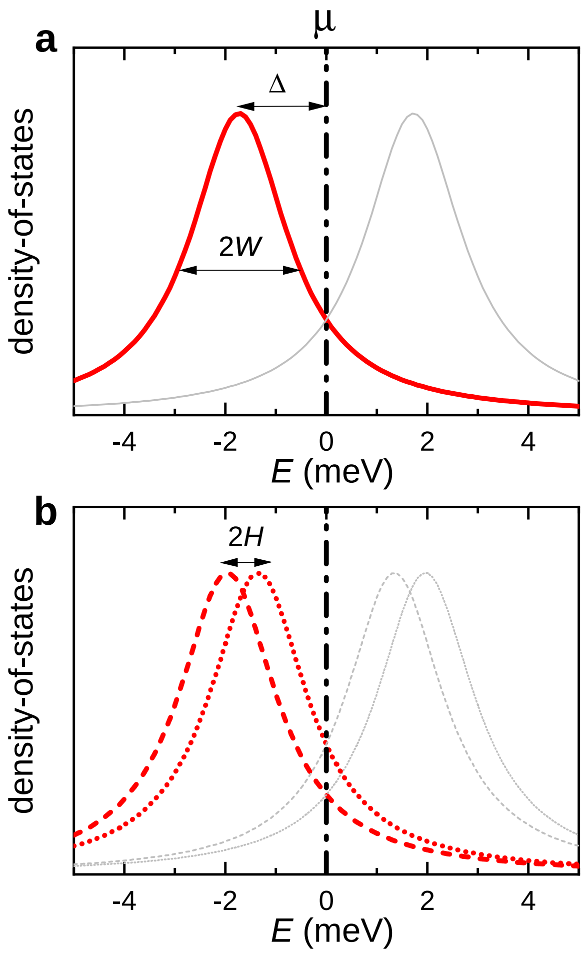

Supplementary Figure 6: Electronic density-of states according to the Schotte-Schotte model. **a**, Schematic Lorentzian-shaped electronic density-of-states according to the Schotte-Schotte mode (red thick line).[1] In the fit, we cannot rule out the existence of a similar feature located at positive energies (grey thin line).[2] The vertical black dot-dashed line indicates the location of the chemical potential μ at which $\varepsilon = 0$. **b**, The same bands (red dashed and dotted curves) split by a Zeeman splitting energy $2H = g_{\text{eff}}\mu_{\text{B}}B$.

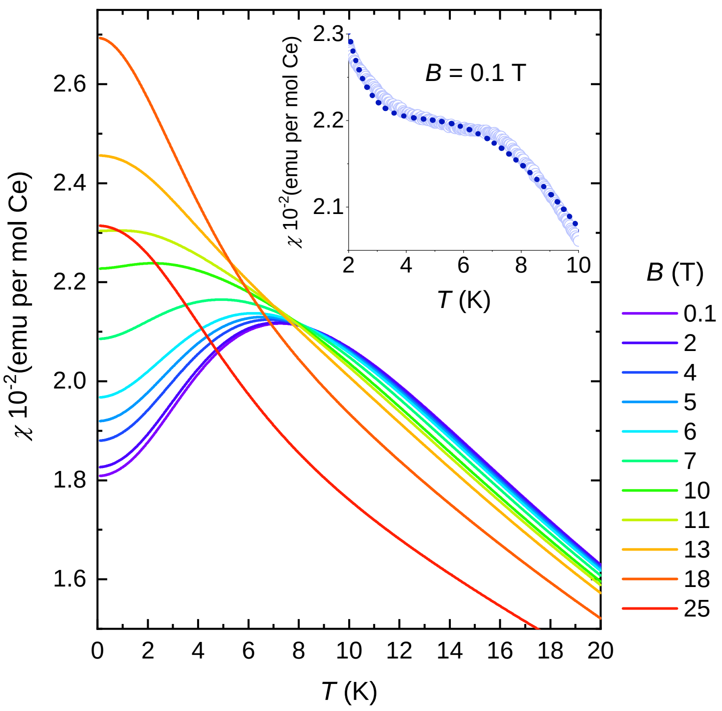

Supplementary Figure 7: Simulations of the temperature and magnetic field-dependent magnetic susceptibility of $Ce_3Bi_4Pd_3$. Calculated susceptibility according to Equation (3) of the main text, using $\Delta = 1.5$ meV and $g_{\text{eff}} = 2.7$, at different magnetic fields as indicated in different colors. The inset shows a dotted line fit to the experimentally measured susceptibility at 0.1 T including an additional Curie-Weiss term in order to account for the presence of impurities.

Supplementary References

[1] Schotte, K. D., Schotte, U., Interpretation of Kondo experiments in a magnetic field. *Phys. Lett.* **55A**, 38-40 (1975).

[2] Tomczak, J. M., Thermoelectricity in correlated narrow-gap semiconductors. *J. Phys.: Condens. Matter* **30**, 183001 (2018).
